# Validation of the test for finding word retrieval deficits (WoFi) in detecting Alzheimer's disease in a naturalistic clinical setting

**DOI:** 10.1007/s10433-023-00772-z

**Published:** 2023-06-30

**Authors:** Eleni-Zacharoula Georgiou, Maria Skondra, Marina Charalampopoulou, Panagiotis Felemegkas, Asimina Pachi, Georgia Stafylidou, Dimitrios Papazachariou, Robert Perneczky, Vasileios Thomopoulos, Antonios Politis, Iracema Leroi, Polychronis Economou, Panagiotis Alexopoulos

**Affiliations:** 1 Mental Health Services, Patras University General Hospital, Department of Medicine, School of Health Sciences, University of Patras, Patras, Greece; 2 Department of Speech and Language Therapy, School of Health Rehabilitation Sciences, University of Patras, Patras, Greece; 3 Department of Philology, School of Humanities and Social Sciences, University of Patras, Patras, Greece; 4 Division of Mental Health in Older Adults and Alzheimer Therapy and Research Center, Department of Psychiatry and Psychotherapy, University Hospital, Ludwig-Maximilians-Universität Munich, Munich, Germany; 5 Ageing Epidemiology (AGE) Research Unit, School of Public Health, Faculty of Medicine, The Imperial College of Science, Technology and Medicine, London, UK; 6 German Center for Neurodegenerative Diseases (DZNE) Munich, Munich, Germany; 7 Munich Cluster for Systems Neurology (SyNergy), Munich, Germany; 8 Sheffield Institute for Translational Neurosciences (SITraN), University of Sheffield, Sheffield, UK; 9 Large-Scale Machine Learning and Cloud Data Engineering Laboratory (ML@Cloud-Lab), Department of Computer Engineering and Informatics, School of Engineering, University of Patras, Patras, Greece; 10 First Department of Psychiatry, Eginition Hospital, School of Medicine, National and Kapodistrian University of Athens, Athens, Greece; 11 Department of Psychiatry, Division of Geriatric Psychiatry and Neuropsychiatry, Johns Hopkins Medical School, Baltimore, USA; 12 Global Brain Health Institute, Medical School, Trinity College Dublin, The University of Dublin, Dublin, Republic of Ireland; 13 Department of Civil Engineering (Statistics), School of Engineering, University of Patras, Patras, Greece; 14 Department of Psychiatry and Psychotherapy, Klinikum rechts der isar, Faculty of Medicine, Technical University of Munich, Munich, Germany; 15 Patras Dementia Day Care Centre, Patras, Greece

**Keywords:** Dysnomia, Auditory stimuli-based naming test, Mild and major neurocognitive disorder

## Abstract

**Background:**

Detecting impaired naming capacity contributes to the detection of mild (MildND) and major (MajorND) neurocognitive disorder due to Alzheimer’s disease (AD). The Test for Finding Word retrieval deficits (WoFi) is a new, 50-item, auditory stimuli-based instrument.

**Objective:**

The study aimed to adapt WoFi to the Greek language, to develop a short version of WoFi (WoFi-brief), to compare the item frequency and the utility of both instruments with the naming subtest of the widely used Addenbrooke’s cognitive examination III (ACEIIINaming) in detecting MildND and MajorND due to AD.

**Methods:**

This cross-sectional, validation study included 99 individuals without neurocognitive disorder, as well as 114 and 49 patients with MildND and MajorND due to AD, respectively. The analyses included categorical principal components analysis using Cramer’s V, assessment of the frequency of test items based on corpora of television subtitles, comparison analyses, Kernel Fisher discriminant analysis models, proportional odds logistic regression (POLR) models and stratified repeated random subsampling used to recursive partitioning to training and validation set (70/30 ratio).

**Results:**

WoFi and WoFi-brief, which consists of 16 items, have comparable item frequency and utility and outperform ACEIIINaming. According to the results of the discriminant analysis, the misclassification error was 30.9%, 33.6% and 42.4% for WoFi, WoFi-brief and ACEIIINaming, respectively. In the validation regression model including WoFi the mean misclassification error was 33%, while in those including WoFi-brief and ACEIIINaming it was 31% and 34%, respectively.

**Conclusions:**

WoFi and WoFi-brief are more effective in detecting MildND and MajorND due to AD than ACEIIINaming.

**Supplementary Information:**

The online version contains supplementary material available at 10.1007/s10433-023-00772-z.

## Introduction

The clinical phenotype of Alzheimer’s disease (AD), being the most common cause of dementia, includes dysnomia. Dysnomia refers to the impairment of naming capacity (Moayedfar et al. 2021; Georgiou et al. 2022). Gradual progressive impairment in lexical retrieval is one of the main symptoms of the early stages of AD, which also encompass decline in memory, attention/concentration, orientation, visuospatial abilities and executive function (Jokel et al. 2019; Jarema et al. 2020; Moayedfar et al. 2021; Knopman et al. 2021). In moderate to severe stages of major neurocognitive disorder (MajorND) due to AD, deficits in verbal fluency, comprehension and literal and semantic paraphrases become prominent, while in very severe AD speech can be restricted to echolalia and verbal stereotypy (Ferris and Farlow 2013; Sachdev et al. 2014). Thus, detection of dysnomia in AD, particularly in the early stages of the disease course, may be crucial in the diagnostic workup of the disease not only in clinical but also in research settings.

Most naming tests which have been validated in patients with neurocognitive disorders are based on visual stimuli (Georgiou et al. 2022). Nonetheless, visual perceptual problems are relatively common in aging and prominent in some neurocognitive disorders like the Lewy Body Disorders and may confound the findings of visual naming tests (Hirsch et al. 2021). In addition, visual naming tests are not useful in distinguishing anomia from visual agnosia, an impairment found in sequelae of stroke and in various neurodegenerative conditions such as posterior cortical atrophy (Schott and Crutch 2019; Heutink et al. 2019). Auditory naming tests may bypass biases stemming from visual perceptual deficits and were shown to have a greater validity in detecting very mild word-finding difficulties compared to visual ones (Hirsch et al. 2016, 2021).

The test for finding word retrieval deficits (Word Finding = WoFi) is a newly formed naming assessment tool, created for the purpose of detecting dysnomia in early stages of AD (Camerer-Waldecker and Supprian 2019). WoFi is a non-visual naming test. Its administration time does not exceed 15 min. WoFi consists of 50 items, most of which are three-syllable simplicia. Regarding the frequency of use of WoFi items in the German language, 5% of WoFi items are very rarely used words, 60% are rare, 30% are infrequent, and 5% are frequent (Camerer-Waldecker and Supprian 2019), resulting in an average test item frequency lower than that of all naming tests that have been validated in neurocognitive disorders so far (Georgiou et al. 2022). Less frequent words are in general more difficult to name (Yonelinas 2002), while word frequency seems particularly to influence naming success of older adults with AD (Thompson-Schill et al. 1999; Gale et al. 2009). According to the findings of the initial validation study which included 20 cognitively healthy older adults and 40 patients with mild to moderate MajorND due to AD, WoFi has an excellent accuracy (95% sensitivity, 92% specificity) (Camerer-Waldecker and Supprian 2019). Nonetheless, it has not been validated in mild neurocognitive disorder (MildND), which is a clinical entity existing between healthy cognitive aging and MajorND, and the detection of which is not always a straightforward process (Albert et al. 2011; Saunders et al. 2022).

The aims of the study were (i) to adapt WoFi to the Greek language; (ii) to develop a brief, time feasible version of WoFi (WoFi-brief) encompassing the items of the original WoFi which safeguard comparable accuracy in recognizing AD with the original WoFi in Greek-speaking adults; (iii) to compare the utility and item frequency of both WoFi and WoFi-brief in detecting MildND and MajorND due to AD with that of the naming subtest of the widely used Addenbrooke’s cognitive examination III (ACEIIINaming) (Calderón et al. 2021) in a naturalistic clinical setting.

## Materials and methods

### Participants

The study encompassed adults who underwent a diagnostic workup between 2019 and 2022 at the old-age mental health outpatient clinic of the Patras University General Hospital in Western Greece. The study was conducted in accordance with the latest revision of the Declaration of Helsinki and was approved by the hospital bioethics and research ethics committee. All participants or authorized representatives gave their written informed consent after a thorough description of the study aims and protocol and prior to study enrollment. Inclusion criteria for the entire sample were (1) (self-) referral for diagnostic evaluation due to cognitive complaints and/or family concerns related to symptoms of neurocognitive disorders or within the frames of preventive cognitive health checks, (2) age ≥ 45 years, (3) diagnosis of MildND or MajorND due to AD, or absence of a neurocognitive disorder. Exclusion criteria were (1) diagnosis of a neurocognitive disorder caused by a disease other than AD (e.g., frontotemporal lobar degeneration, Parkinson’s disease), (2) mental or neurological disorder or unstable medical condition potentially affecting cognitive function (e.g., major depression, schizophrenia, multiple sclerosis, seizure disorder, head injury, uncontrolled hypothyroidism), (3) uncorrected, severe hearing or visual difficulties, being potential sources of bias in diagnostic accuracy, (4) insufficient knowledge of the Greek language and (5) unwillingness to participate in the study.

Clinical diagnoses relied on the findings of a thorough diagnostic workup and were established according to international diagnostic criteria. The diagnostic assessment included a history from the examinee and from an informant; neurological and psychiatric examination; laboratory screening and brain imaging (CT or MRI), provided cognitive impairment was detected, and the administration of the Mini-Mental State Examination (MMSE) (Kourtesis et al. 2020), the Montreal Cognitive Assessment (MoCA) (Dautzenberg et al. 2021) and the Cognitive Telephone Screening Instrument plus (COGTEL +) (Alexopoulos et al. 2021), assessing a relatively wide range of cognitive domains. The diagnosis of MildND and MajorND due to AD was based on the DSM-5 diagnostic criteria (Sachdev et al. 2014) and on the diagnostic guidelines of the National Institute on Aging-Alzheimer Association (Albert et al. 2011; McKhann et al. 2011). In individuals without cognitive impairment, neither cognitive deficits nor functional impairment was detected. The clinician who established the diagnoses was blind to the individual performance on the naming tests.

### Ethical considerations

The study was conducted in accordance with the latest revision of the Declaration of Helsinki and was approved by the hospital bioethics and research ethics committee. All participants or authorized representatives gave their written informed consent after a thorough description of the study aims and protocol and prior to study enrollment.

### Neurocognitive instruments

Based on the advice of its developers, Wo-Fi was translated into Greek and afterward a bilingual clinician not familiar with the original version of the instrument performed a back-translation into German. Ten items were replaced because of the presence of synonyms in nine cases and due to the necessary inclusion of a word in the respective question which had as stem the morpheme #pjan#, as did the correct answer (“How do we call the person who professionally plays the piano?”, “Pianist”). These items were replaced by words of the same frequency, which do not have synonyms in Greek (Kilgarriff et al. 2004, 2014) (Additional File 1: Table S1). The comparison of the original German version to the version derived from the back-translation process showed that the new version was similar to the original one except for the adjusted items. Of note, the average number of words per question was lower in the Greek version compared to the original one (11.9 words/question vs. 22.4 words/question, respectively). The Greek version was scored by assigning one point for each correct answer and has a maximum score of 50 points. Like the German original, the Greek Wo-Fi can be administered in approximately 15 min.

The Addenbrooke’s cognitive examination III, being a widely used brief instrument, assesses naming ability with a subtest including twelve line drawings (ACEIIINaming) (Kourtesis et al. 2020). The average test item frequency of its English version was classified as infrequent (Georgiou et al. 2022). A recent report on the psychometric properties of the ACE-III, which relied on an item response theory approach, unveiled that ACEIIINaming has an adequate goodness-of-fit, both to item and model levels, and its items contribute to discriminating between MajorND due to AD and cognitively healthy older adults (Calderó et al. 2021).

### Naming test item frequency

The three naming tests were compared regarding the frequency of the words each one of them includes. The assessment of the frequency of each item was based on corpora of television subtitles, which are considered one of the best measures of word frequency (Brysbaert et al. 2018). SUBTLEXWF is the frequency per million words (subtitle frequency: word form frequency) (Brysbaert and New 2009). The Greek frequency database SUBTLEX-GR, a corpus with over 23 million Modern Greek words, was employed (Dimitropoulou et al. 2010). The metric FREQcount, i.e., the number of times the word appears in the corpus (raw frequency), was used. Checked thoroughly against the database, the frequency of each item of the naming tests was then classified into one of the following frequency categories: 1: rare, 2: infrequent, 3: frequent, 4: very frequent (Georgiou et al. 2022). For the classification of naming test items into these arbitrary chosen four frequency categories, the words of SUBTLEX-GR database were ordered by frequency count and then the first 25% were determined to have rare frequency, the next up to 50% to be infrequent, etc. For each test a mean total score of frequency categories was calculated based on the ratio of the sum of the frequency category of all test items divided by the total number of test items.

### Statistical analyses

As the number of WoFi items is relatively high, a heuristic approach was adopted to select a subset of questions forming the WoFi-brief without however severely impairing the utility of the brief version compared to the original one. Initially, items with the same pattern of distribution of correct vs. erroneous answers across the three diagnostic groups were excluded from further investigation as they did not unveil differences in cognitive function between older adults with and without cognitive impairment due to AD. For the remaining items the categorical principal components analysis using Cramer’s V Correlation as a measure of the strength of the relationship between the nominal variables/items (Meulman et al. 2002; Linting et al. 2007) was employed not as a method of dimension reduction but rather as an auxiliary, heuristic approach of feature selection by revealing relationships among variables (Lu et al. 2007; Song et al. 2010). For each one of the first k principal components (PC) (the number k of PC was determined so that the percentage of variance captured by these PCs was larger than 80% of the original variables/items), the item with the largest loading was selected. Additional items were selected from each principal component, provided their loading was larger than the minimum of the maximum loadings in the k principal components.

Pairwise comparisons and differences across the three study groups, i.e., individuals without cognitive impairment, patients with MildND or MajorND due to AD, in sex distribution, age, education and test scores, were assessed with Pearson Chi-square test, Kruskal–Wallis Chi-square test and pairwise test of proportions or Dunn test for post hoc multiple comparisons, as appropriate, since data normality assumption was rejected based on analysis of skewness and kurtosis. Three proportional odds logistic regression models (POLR models) were employed for studying the relationship between diagnostic groups (served as the ordinal dependent variable) and each one of the three different instruments (WoFi, WoFi-brief and ACEIIINaming) taking into account age, sex and education, which influence cognitive function in older adults (Bernardelli et al. 2020). Stratified repeated random subsampling (stratified bootstrap resampling) was used to recursive partitioning to training and validation set (70/30 ratio) (James et al. 2005; Lokhov et al. 2012; Alexopoulos et al. 2021; Skarlatos et al. 2023). The procedure was repeated 20,000 times, and the results (parameters estimates over the training data sets and misclassification errors over the training and the validation data sets) were then averaged over the splits. Kernel Fisher discriminant analysis models were also employed to compare the capacity of the three instruments to separate correctly the three study groups (Maciej Serda et al. 1999; Baudat and Anouar 2000).

## Results

### Demographic of study groups

The study sample encompassed 99 individuals without cognitive impairment (WCO), 114 patients with MildND and 49 with MajorND due to AD (Table 1). Age (KW Chi-square = 41.11, p < 0.001, df = 2) and education (KW Chi-square = 49.39, *p* < 0.001, df = 2) significantly differed between the groups, while sex distribution did not (Pearson Chi-square = 2.51, df = 2 *p* = 0.29, df = 2) (Table 1).

**Table 1:** Demographic, neurocognitive and clinical characteristics of the study sample

	Individuals without neurocognitive disorder (Group 1, G1)	Mild neurocognitive disorder due to Alzheimer’s disease (Group 2, G2)	Major neurocognitive disorder due to Alzheimer’s disease (Group 3, G3)	Pairwise comparisons		
				G1 vs. G2	G1 vs. G3	G2 vs. G3
N	99	114	49			
Age, years*	65.3 (9.36) [45–83]	72.3 (7.56) [46–86]	74.6 (10.0) [56–92]	< 0.001^‡^	< 0.001^‡^	0.69^‡^
Education, years*	12.8 (3.46) [5–18]	9.71 (4.13) [3–18]	7.84 (4.41) [0–18]	< 0.001^‡^	< 0.001^‡^	0.07^‡^
Sex (female)	70 [71%]	71 [62%]	29 [59%]	0.672^†^	0.672^†^	0.844^†^
MOCA*	27.5 (1.88) [19–30]	22.6 (3.45) [10–29]	14.2 (4.79) [3–24]	< 0.001^‡^	< 0.001^‡^	< 0.001^‡^
MMSE*	29.1 (1.07) [26–30]	26.3 (2.51) [13–30]	18.4 (5.14) [3–29]	< 0.001^‡^	< 0.001^‡^	< 0.001^‡^
COGTEL + *	35.4 (9.44) [0–58]	22.9 (6.55) [6–36.9]	11.1 (6.15) [0–29]	< 0.001^‡^	< 0.001^‡^	< 0.001^‡^
WoFi*	45.8 (5.17) [19–50]	36.0 (9.26) [10–50]	21.5 (13.7) [0–50]	< 0.001^‡^	< 0.001^‡^	< 0.001^‡^
WoFi-brief*	14.0 (2.27) [7–16]	9.84 (3.84) [0–16]	4.84 (4.23) [0–16]	< 0.001^‡^	< 0.001^‡^	< 0.001^‡^
ACEIIINaming*	11.7 (0.859) [8–12]	9.81 (2.64) [2–12]	6.65 (3.50) [0–12]	< 0.001^‡^	< 0.001^‡^	< 0.001^‡^

^*^mean (standard deviation) [range]

*MOCA* Montreal Cognitive Assessment, *MMSE* Mini-Mental State Examination, *COGTEL* + Cognitive Telephone Screening Instrument plus six orientation items of the Montreal Cognitive Assessment, *WoFi* Test for finding word retrieval deficits, *WoFi-brief* Test for finding word retrieval deficits brief version, *ACEIIINaming* Addenbrooke’s cognitive examination III naming subtest

^‡^ Dunn test for post hoc multiple comparisons after Kruskal–Wallis Chi-square test; ^†^pairwise test of proportions after Pearson Chi-square test

### Creation of WoFi-brief

Based on the lack of difference in the pattern of the distribution of correct vs. erroneous answers across the three diagnostic groups, 20 items were excluded (Additional File 1: Fig. S1). In addition, the categorical principal components analysis revealed 15 principal components which explained more than 80% of the variance of the original data. Thus, a set of 15 items was selected. An additional item was also selected from the first principal component as its loading was larger than the minimum of the maximum loading in the first 15 principal components, resulting in a set of 16 items which form the WoFi-brief (Additional File 1: Fig. S1, Table S2). The average number of words per question in the new version of the instrument is 8.75. The duration of WoFi-brief administration does not exceed five minutes.

**Fig. 1:**
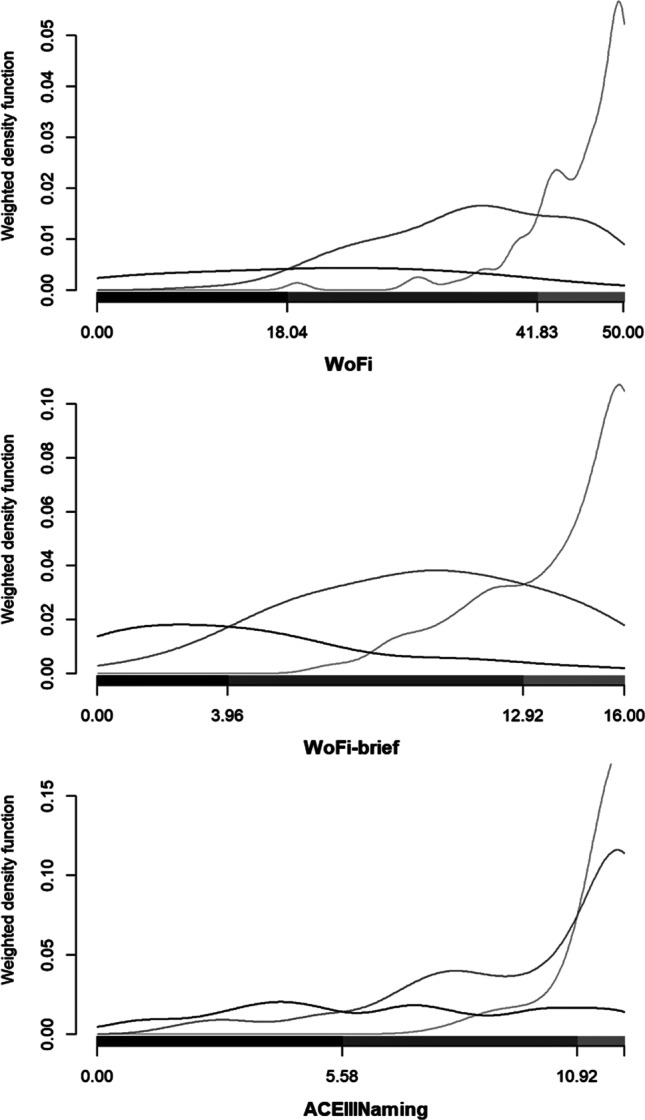
Weighted density function, scores on the word-finding retrieval test (WOFI), the brief version of WoFi (WoFi-brief) and the naming items of the Addenbrooke’s Cognitive Examination III (ACEIIINaming) and cutoff points for diagnosing mild and major neurocognitive disorder due to Alzheimer’s disease

### Test item frequency

The average item frequencies of WoFi and WoFi-brief were clearly lower compared to ACEIIINaming. The mean FREQcount (standard deviation [minimum–maximum]) was 946.96 (2139.45 [0–98.40]) and 762 (1736.20 [10–6580]) of items of WoFi and WoFi-brief, respectively. The mean FREQcount of ACEIIINaming was 3760.83 (11,145.3 [10–39090]). The sum of the frequency categories of all test items divided by the total number of test items was 3.2 for both WoFi and WoFi-brief, while it was higher for ACEIIINaming (3.42).

### Cognitive data of study groups

Performance on MOCA (KW Chi-square = 179.42, *p* < 0.001, df = 2), MMSE (KW Chi-square = 170.41, *p* < 0.001, df = 2), COGTEL + (KW Chi-square = 160.61, *p* < 0.001, df = 2), WoFi (KW Chi-square = 120.58, *p* < 0.001, df = 2), ACEIIINaming (KW Chi-square = 96.34, *p* < 0.001, df = 2) and the newly designed WoFi-brief (KW Chi-square = 120.91, *p* < 0.001, df = 2) significantly differed between the groups. Results of pairwise comparison analyses are presented in Τable 1.

Three POLR models were employed to investigate the relationship between diagnostic status (0: no cognitive impairment, 1: MildND, 2: MajorND) and performance on each one of the studied instruments WoFi, WoFi-brief and ACEIIINaming. The three POLR models have been incorporated into the following Google sheet and can be used for estimating the probability of an individual to belong to one of the three diagnostic categories (no cognitive impairment, MildND, MajorND due to AD) according to her/his performance on naming tests and demographic characteristics (http://www.des.upatras.gr/amm/economou/NeurocognitiveAssessment.html). In Table 2 the averages of the parameters of the three models along with their 95% bootstrap confidence intervals based on 20,000 stratified bootstrap training sets are presented. Performance on dysnomia test was significantly related to diagnostic category in all three models. As expected, higher performance on the studied cognitive instruments pertained to less severe diagnostic category in all models. Age significantly pertained to the diagnostic group in all three models. For example, holding everything else constant, an increase in age by one year increases the expected value of diagnostic status in log odds by 0.041 according to the findings of the POLR model which included WoFi-brief as an independent variable. Education was significantly associated with the diagnostic category only in the models which included WoFi and ACEIIINaming, while sex was related to diagnostic status only in the model that encompassed ACEIIINaming, since the 5% bootstrap confidence intervals in all other cases contained zero (Table 2).

**Table 2:** The averages of the parameters of the three proportional odds logistic regression models along with their 95% bootstrap confidence intervals based on 20,000 stratified bootstrap training sets

		POLR including WoFi as independent variable	POLR including WoFi-brief as independent variable	POLR including ACEIIINaming as independent variable
95% bootstrap confidence intervals	y > = 2	5.08097 [1.0653] (3.06115, 7.22959)	2.94423 [0.99854] (1.04457, 4.94541)	4.68160 [1.0840] (2.61507, 6.88026)
	y > = 3	1.60011 [1.02510] (− 0.40095, 3.62435)	− 0.60003 [0.97622] (− 2.51128, 1.31241)	1.55011 [1.05124]–(0.49467, 3.64619)
Covariates	Age	0.03196 [0.01103] (0.01074, 0.05414)	0.04098 [0.01087] (0.02022, 0.06298)	0.03692 [0.01101] (0.01620, 0.05929)
	Sex	− 0.25181 [ 0.18910] (− 0.62650, 0.11398)	− 0.34545 [0.19255] (− 0.72861, 0.02678)	0.46588 [0.18402] (− 0.83751, − 0.11483)
	Education	− 0.05829 [ 0.02453] (− 0.10641, − 0.01046)	− 0.03299 [0.02510] (− 0.08305, 0.01710)	− 0.15143 [0.02250] (− 0.19769 − 0.10965)
	Neurocognitive instrument	− 0.14242 [0.01176] (− 0.16746, − 0.12205)	− 0.37367 [0.02962] (− 0.43710, − 0.32071)	− 0.40748 [0.03508] (− 0.48269, − 0.34533)

*POLR* Proportional odds logistic regression model, *WoFi* Test for finding word retrieval deficits, WoFi-brief: Test for finding word retrieval deficits brief version, *ACEIIINaming* Addenbrooke’s cognitive examination III naming subtest

Differences in misclassification errors between WoFi, WoFi-brief and ACEIIINaming were unveiled. According to the results of the discriminant analysis, the cutoff values for detecting MildND and MajorND due to AD were 41.83 and 18.04 for WoFi, 12.92 and 3.96 for WoFi-brief and 10.92 and 5.58 for ACEIIINaming (Fig. 1). The misclassification error was 30.9%, 33.6% and 42.4% for WoFi, WoFi-brief and ACEIIINaming, respectively. The POLR models including WoFi and WoFi-brief as dependent variables outperformed the models with ACEIIINaming as dependent variable. Misclassification errors (%) over the 20,000 stratified bootstrap training and validation sets along with their 95% bootstrap confidence intervals are presented in Table 3. WoFi-brief was found to have the lowest average misclassification error in both the training- and the validation data set. In all cases the average misclassification error varied between 30 and 35%.

**Table 3:** The mean misclassification errors (%) of the three proportional odds logistic regression models along with their 95% bootstrap confidence intervals based on 20,000 stratified bootstrap training and validation sets

	Training sets	Validation sets
POLR including WoFi as independent variable*	0.3109 (0.2732, 0.3497)	0.3262 (0.2405, 0.4177)
POLR including WoFi-brief as independent variable*	0.3015 (0.2623, 0.3443)	0.3139 (0.2278, 0.4050)
POLR including ACEIIINaming as independent variable*	0.3291 (0.2896, 0.3716)	0.3432 (0.2532, 0.4304)

*POLR* Proportional odds logistic regression mode, *WoFi* Test for finding word retrieval deficits, *WoFi-brief* Test for finding word retrieval deficits brief version, *ACEIIINaming* Addenbrooke’s cognitive examination III naming subtest

## Discussion

The present study has demonstrated the utility of the new, auditory stimuli- based dysnomia tests WoFi and WoFi-brief in identifying MildND and MajorND due to AD. Compared to WoFi, WoFi-brief is shorter but equally effective in detecting naming deficits and subsequently more feasible in clinical settings. The novelty of the study comprises (1) the development of WoFi-brief, (2) the inclusion of a relatively large and well-characterized sample of patients with MildND, (3) the naturalistic study design based on individuals referred for cognitive concerns to a university hospital-based, old-age psychiatry outpatient clinic, as well as (4) the direct comparison of WoFi and WoFi-brief with ACEIIINaming subtest, which is part of the widely used ACE-III (Kourtesis et al. 2020). We found significant associations between performance on all three considered naming tools and diagnostic status. Lower scores were shown to pertain to phenotypes characterized by more severe cognitive deficits.

WoFi and WoFi-brief outperformed ACEIIINaming in detecting both MildND and MajorND due to AD. Both WoFi and WoFi-brief exhibited lower misclassification errors compared to the dysnomia subtest of the ACEIIINaming according to the findings of the POLR models (WoFi and WoFi-brief: 30.2–32.7% vs. ACEIIINaming: 32.9–34.3%) and of the discriminant analyses (WoFi and WoFi-brief: 30.9–33.6% vs. ACEIIINaming: 42.4%). This is not unexpected, since the German version of WoFi has the lowest average test item frequency when all naming tests that have been validated in neurocognitive disorders are considered (Georgiou et al. 2022). According to our analyses, the Greek version of WoFi and WoFi-brief has lower mean item frequency compared to ACEIIINaming. Moreover, auditory naming tests were shown to bypass biases stemming from visual perceptual deficits and to have higher validity in detecting very mild word-finding difficulties (Hirsch et al. 2016; Salehi et al. 2017). In addition, the higher utility of WoFi and WoFi-brief compared to ACEIIINaming may be attributed to the fact that auditory naming is more naturalistic, since it is more strongly correlated with the context in which word-finding impairment is usually expressed in real life, i.e., through dialog and interpersonal conversations rather than as difficulties in naming of drawings (Georgiou et al. 2022).

WoFi-brief seems to have a slightly better classificatory utility compared to WoFi. The development of a brief version of WoFi, which has comparable or even slightly better utility in detecting MildND and MajorND due to AD compared to the original one, may catalyze the incorporation of this brief tool into routine diagnostic procedures not only of secondary and tertiary brain healthcare services, but also into primary healthcare services, in which time feasibility of the diagnostic workup of cognitive complaints is vital for both the clinician and the examinee (Georgiou et al. 2022). In contrast to POLR models, the findings of the discriminant analysis point a higher validity of WoFi compared to its shorter version. This result may be attributed to the fact that in discriminant analyses demographic data were not considered, even though age significantly pertained to diagnostic status in the models including WoFi and WoFi-brief and education was found to be related to diagnostic status in the models with WoFi (Table 2). Despite the higher time feasibility of WoFi-brief, the original WoFi may be more suitable for tracking changes in naming ability over time not only in individuals who do not yet fulfill the criteria for neurocognitive disorders but complain about cognitive decline, but also in symptomatic AD. Particularly in the former group, detection of subclinical decline may signal AD or other dementia risk and pave the way toward causal therapies in countries in which they are available (Langhough Koscik et al. 2021; Larkin 2023).

Even though a naming test should not be a vocabulary test (Hamberger 2015), WoFi and WoFi-brief have an average frequency of words lower than ACEIIINaming. It is known that low-frequency items elicit errors in examinees with limited education and vocabulary due to lack of familiarity, while highly frequent words are named correctly by virtually all, resulting in low sensitivity (Hamberger et al. 2022). The influence of education in the case of WoFi points to difficulties in eliminating the influence of vocabulary in the process of developing a valid naming test. Compared to mean total score of frequency categories of the German version of WoFi, the Greek WoFi consists of more frequently used items (German WoFi: 1.2 vs. Greek WoFi: 3.2), as ACEIIINaming does (2 in English vs. 3.42 in Greek) (Georgiou et al. 2022). This change in average total scores of frequency categories is attributable to the accurate translation of most of the items of the original, German WoFi into Greek. Nevertheless, the frequency of words varies across languages (Tjuka 2020). Implementing an adaptation strategy of WoFi into the Greek language exclusively based on word frequency would have resulted in a test only marginally related to the original one.

The relatively high misclassification errors of all considered naming tests were hardly unexpected. The gold standard of the analytical endeavors in this study was the clinical diagnoses of MildND or MajorND due to AD or their absence, and not the presence or absence of naming deficits, which are common but not ubiquitously present particularly in early AD (Silagi et al. 2015). Thus, the far from being excellent detected misclassification errors can be easily interpreted. Of note, widely used, established instruments in the diagnostic workup of neurocognitive disorders were shown to have misclassification errors higher than those detected here (Beishon et al. 2019; Ratcliffe et al. 2022).

WoFi and WoFi-brief, being auditory stimuli-based naming tests, can be administered over the telephone in constellations in which face-to-face assessment is not feasible or appropriate. Tele-neurocognitive assessment enables frequent, less time-consuming and more cost-effective monitoring of cognition within the frames of longitudinal studies and mental telehealth services (Sorinmade et al. 2020; Karamanis et al. 2022). Older people are more familiar with telephone compared to the more complex videoconferencing, may have low technical readiness and trust in technologies and might therefore face barriers to the effective use of digital health tools (Lattie et al.; Dequanter et al. 2022; Mahmoud et al. 2022). Thus, WoFi and WoFi-brief may be valuable for assessing cognitively frail older adults who live in remote communities or in middle- and low-income countries (Lattie et al.; Mahmoud et al. 2022) or cannot easily access mental and cognitive healthcare services.

The present study has a number of limitations. First, the evaluation was confined to patients with MildND and MajorND due to AD. Hence, we were not in the position to assess the effectiveness of WoFi and WoFi-brief in detecting neurocognitive disorder caused by other diseases, such as Lewy bodies or cerebrovascular pathologies. Moreover, an automatically, i.e., without prompt, corrected answer was scored with one point, as did correct answers, while according to the scoring guidelines of the German version of WoFi, two points are assigned for correct answers, one for an automatically corrected answer and zero points for erroneous answers (Camerer-Waldecker and Supprian 2019). Our approach, being in line with most neurocognitive instrument scoring guidelines which score examinees’ answers as either correct or false, may bypass potential bias stemming from the complex interplay between personality traits, non-cognitive symptoms of neurocognitive disorders (e.g., impulsivity) and cognitive and functional performance (Rouch et al. 2019; Sakurai et al. 2020; Cerni et al. 2021; Giannakis et al. 2021). Furthermore, it may be reckoned that differences in the length of questions of the two here studied auditory stimuli-based dysnomia instruments may have biased our observations since individuals with cognitive impairment face difficulties in understanding long sentences (van Boxtel and Lawyer 2021). In such a case, low performance on WoFi and WoFi-brief may be attributed not only to dysnomia but also to comprehension difficulties. Nevertheless, the two tools were comparably effective in detecting MildND and Major ND, even though the average number of words per question was lower in WoFi-brief compared to WoFi, indicating that the questions of the former may be more easily understood by individuals with cognitive impairment. In addition, the group of participants without neurocognitive disorder included not only people with subjective cognitive complaints which may contribute to misdiagnosis of MildND and are associated with an increased risk of incident MildND and MajorND, but also individuals who were assessed within the frames of preventive cognitive health checks. Nonetheless, all people included in this study group did not fulfill the respective diagnostic criteria for either MildND or MajorND (Edmonds 2014; van Harten et al. 2018). Finally, the clinical diagnosis, which was based on a comprehensive diagnostic procedure and on international diagnostic criteria, was used as the ultimate gold standard. Since the clinical diagnoses are neither always confirmed at autopsy nor always supported by biomarker constellations typical for AD, possibly erroneous clinical assessments should be also taken into account (Alexopoulos et al. 2016, 2018; Degenhardt et al. 2016). The validity of WoFi and WoFi-brief may therefore be lower than our results suggest.

Overall, this study has demonstrated the clinical utility of WoFi and its short version WoFi-brief in detecting not only MajorND due to AD but also MildND. Both instruments can be employed in variable settings, meeting the needs of clinicians for valid tests assessing naming ability in older adults with cognitive complaints. The administration of WoFi and WoFi-brief over the telephone may extend their usefulness, since they may prove useful in large-scale cross-sectional and longitudinal aging studies and mental telehealth services.

## Supplementary Information

Below is the link to the electronic supplementary material.

**Additional file 1**: The Greek version of the Test for Finding Word retrieval deficits (WoFi), its brief version (WoFi-brief) and the distribution of correct vs. erroneous answers to each item of WoFi across the three study groups.

## Data Availability

Data of the study are available from the corresponding author on reasonable request.
