# Supplementary material for: Validation of the test for finding word retrieval deficits (WoFi) in detecting Alzheimer's disease in a naturalistic clinical setting
Source: Eur J Ageing. 2023 Jun 30;20(1):29. doi: 10.1007/s10433-023-00772-z (PMC10313575; doi:10.1007/s10433-023-00772-z)
Supplement: Supplementary file 1 — Additional file 1: The Greek version of the Test for Finding Word retrieval deficits (WoFi), its brief version (WoFi-brief) and the distribution of correct vs. erroneous answers to each item of WoFi across the three study groups. [file 10433_2023_772_MOESM1_ESM.docx]

**Validation of the Test for Finding Word retrieval deficits (WoFi) in detecting Alzheimer's Disease in a naturalistic clinical setting**

Eliza (Eleni-Zacharoula) **Georgiou**^1^, Maria **Skondra**^1^, Marina **Charalampopoulou**^1^, Panagiotis **Felemegkas**^1^, Asimina **Pachi**^1^, Georgia **Stafylidou**^2^, Dimitrios **Papazachariou**^3^, Robert **Perneczky**^4,5,6,8^, Vasileios **Thomopoulos**^9^, Antonios **Politis**^10,11^, Iracema **Leroi**^12^, Polychronis **Economou**^13^, Panagiotis **Alexopoulos**^1,12,14,15^

^1^Department of Psychiatry, Patras University General Hospital, Faculty of Medicine, School of Health Sciences, University of Patras, Patras, Greece

^2^Faculty of Speech and Language Therapy, School of Health Rehabilitation Sciences, University of Patras, Patras, Greece

^3^Department of Philology, School of Humanities and Social Sciences, University of Patras, Greece,

^4^Division of Mental Health in Older Adults and Alzheimer Therapy and Research Center, Department of Psychiatry and Psychotherapy, University Hospital, Ludwig-Maximilians-Universität Munich, Munich, Germany.

^5^Ageing Epidemiology (AGE) Research Unit, School of Public Health, Faculty of Medicine, The Imperial College of Science, Technology and Medicine, London, UK.

^6^German Center for Neurodegenerative Diseases (DZNE) Munich, Munich, Germany.

^7^Munich Cluster for Systems Neurology (SyNergy), Munich, Germany.

^8^Sheffield Institute for Translational Neurosciences (SITraN), University of Sheffield, Sheffield, UK.

^9^Large-Scale Machine Learning and Cloud Data Engineering Laboratory (ML@Cloud-Lab), Faculty of Computer Engineering and Informatics, School of Engineering, University of Patras, Patras, Greece

^10^First Department of Psychiatry, Eginition Hospital, School of Medicine, National and Kapodistrian University of Athens, Athens, Greece.

^11^Department of Psychiatry, Division of Geriatric Psychiatry and Neuropsychiatry, Johns Hopkins Medical School, Baltimore, USA.

^12^Global Brain Health Institute, Medical School, Trinity College Dublin, The University of Dublin, Dublin, Republic of Ireland

^13^Department of Civil Engineering (Statistics), School of Engineering, University of Patras, Patras, Greece

^14^Department of Psychiatry and Psychotherapy, Klinikum rechts der Isar, Faculty of Medicine, Technical University of Munich, Munich, Germany

^15^Patras Dementia day care centre, Patras, Greece

**Τable 1S** Τhe Greek version of the Test for Finding Word retrieval deficits (WoFi)

| **Δοκιμασία Εύρεσης Λέξεων (Δ.Ε.ΛΕ)**  Όνομα: Ηλικία:  Φύλο:  Έτη εκπαίδευσης: Μητρική γλώσσα:  Επάγγελμα: Hμερομηνία:   \|  \| **Eρωτήσεις** \| **Σωστό** \| **Λάθος** \| **Καμιά Απάντηση** \| \| --- \| --- \| --- \| --- \| --- \| \| 1 \| Πως λέμε την ημέρα που γινόμαστε κατά ένα χρόνο μεγαλύτεροι; \|  \|  \|  \| \| 2 \| Πως λέγεται το ζώο που φέρει oπλές, μαύρες και άσπρες ρίγες και βρίσκεται στο Ζωολογικό Κήπο; \|  \|  \|  \| \| 3 \| Πως λέγεται στα ελληνικά ο άνθρωπος που επιβλέπει και ρυθμίζει τη διεξαγωγή ενός αγώνα σύμφωνα με ορισμένους κανονισμούς \|  \|  \|  \| \| 4 \| Πως λέγεται το μαρσιποφόρο ζώο που ζει στην Αυστραλία; \|  \|  \|  \| \| 5 \| Πως λέγεται το σακούλι ενός μεταξοσκώληκα από τον οποίο παίρνουμε το μετάξι; \|  \|  \|  \| \| 6 \| Πως λέγεται η λεπτή γκρι σκόνη, που παραμένει ως κατάλοιπο όταν σβήσει το τσιγάρο; \|  \|  \|  \| \| 7 \| Πως λέγεται το εργαλείο με το οποίο κάνουμε ενέσεις; \|  \|  \|  \| \| 8 \| Πως λέγεται το έντομο χωρίς φτερά που πλέκει ιστό για να πιάνει τα θύματα του; \|  \|  \|  \| \| 9 \| Πως λέγεται το έγγραφο που πιστοποιεί την περάτωση σπουδών στο Γυμνάσιο/Λύκειο; \|  \|  \|  \| \| 10 \| Πως λέγεται η ηλεκτρική συσκευή οικιακής χρήσης για το πλύσιμο ρούχων ή πιάτων; \|  \|  \|  \| \| 11 \| Πως λέγεται το υγρό μέσα σε μια πένα που χρησιμοποιούμε για να γράφουμε; \|  \|  \|  \| \| 12 \| Πως λέγεται το εργαλείο που χρησιμοποιούμε για να καρφώσουμε μια πρόκα στον τοίχο; \|  \|  \|  \| \| 13 \| Πως λέγεται το υγρό υλικό που χρησιμοποιούμε για να ενώσουμε τα σπασμένα κομμάτια ενός βάζου; \|  \|  \|  \| \| 14 \| Πως λέγεται η άκρη της βελόνας μέσα από την οποία περνάμε την κλωστή; \|  \|  \|  \| \| 15 \| Πως λέγεται ο μήνας που γιορτάζουμε τα Χριστούγεννα; \|  \|  \|  \| \| 16 \| Πως λέγεται το καιρικό φαινόμενο που χαρακτηρίζεται από βροχή, αστραπές και βροντές; \|  \|  \|  \| \| 17 \| Πως λέγεται το υλικό από το οποίο είναι κατασκευασμένο ένα λευκό φλιτζάνι του τσαγιού; \|  \|  \|  \| \| 18 \| Πως ονομάζουμε έναν άνθρωπο ο οποίος συνειδητά αποφεύγει να τρώει κρέας; \|  \|  \|  \| \| 19 \| Πώς ονομάζουμε το άτομο που διευθύνει μία ορχήστρα; \|  \|  \|  \| \| 20 \| Πως ονομάζουμε το φυτό που φυτρώνει στην έρημο και έχει αγκάθια; \|  \|  \|  \| \| 21 \| Πως λέμε τον λόγο που βγάζει ένας παπάς στην εκκλησία; \|  \|  \|  \| \| 22 \| Πώς ονομάζουμε το καυτό υλικό που εκτινάσσεται από ένα ηφαίστειο; \|  \|  \|  \| \|  \|  \|  \|  \|  \| \|  \| **Eρωτήσεις** \| **Σωστό** \| **Λάθος** \| **Καμιά Απάντηση** \| \| 23 \| Πως ονομάζουμε το υπόλειμμα του σώματος ενός οργανισμού που διατηρήθηκε ανάμεσα σε πετρώματα παλαιότερων γεωλογικών περιόδων; \|  \|  \|  \| \| 24 \| Πως ονομάζουμε το ζώο που κυνηγάει και τρώει ποντικούς; \|  \|  \|  \| \| 25 \| Πώς ονομάζουμε το υπερυψωμένο τμήμα της εκκλησίας από το οποίο ο παπάς βγάζει λόγο στους πιστούς; \|  \|  \|  \| \| 26 \| Πως λέμε τη νοητή γραμμή που χωρίζει τη γη στο βόρειο και στο νότιο ημισφαίριο; \|  \|  \|  \| \| 27 \| Πως λέμε το νήμα που χρησιμοποιούμε για ράψιμο; \|  \|  \|  \| \| 28 \| Πως λέμε τo πρώτο γράμμα ενός ονόματος και ενός επωνύμου; \|  \|  \|  \| \| 29 \| Πως λέγονται τα χρήματα που καταβάλουμε υποχρεωτικά στο κράτος για την κάλυψη δημοσίων δαπανών ή άλλων αναγκών; \|  \|  \|  \| \| 30 \| Πως λέγεται το αντικείμενο αξίας το οποίο παραδίδει ο δανειζόμενος στο δανειστή του για ασφάλεια του δανείου; \|  \|  \|  \| \| 31 \| Πως ονομάζουμε το κοφτερό μαχαίρι που χρησιμοποιούν οι χειρουργοί; \|  \|  \|  \| \| 32 \| Πως λέγεται το καπέλο/κασκέτο που φορούν σήμερα συνήθως οι ηλικιωμένοι άνδρες και παλιότερα οι εργάτες; \|  \|  \|  \| \| 33 \| Πως ονομάζεται το κόσμημα που στερεώνουν οι γυναίκες στο πέτο της ζακέτας ή του παλτού τους; \|  \|  \|  \| \| 34 \| Πως λέγεται ο δημόσιος λειτουργός που έχει ως έργο του την απονομή της δικαιοσύνης \|  \|  \|  \| \| 35 \| Πως λέγεται η περιουσία που λαμβάνει κάποιος μετά το θάνατο των γονιών του; \|  \|  \|  \| \| 36 \| Πως λέγεται αυτός που γράφει ένα βιβλίο ή ένα μυθιστόρημα; \|  \|  \|  \| \| 37 \| Πως λέμε κάποιον που παίζει ρόλους σε κινηματογραφικές ταινίες; \|  \|  \|  \| \| 38 \| Πως ονομάζουμε την υπερβολική οικονομία χρημάτων; \|  \|  \|  \| \| 39 \| Πως λέγεται κάποιος που είδε μία αξιόποινη πράξη; \|  \|  \|  \| \| 40 \| Πως λέμε το πήλινο ή πλαστικό σκεύος μέσα στο οποίο φυτεύουμε λουλούδια και καλλωπιστικά φυτά; \|  \|  \|  \| \| 41 \| Πως λέγεται το αντίθετο της παλίρροιας; \|  \|  \|  \| \| 42 \| Πως λέγονται οι μακριές τρίχες γύρω από το λαιμό ενός λιονταριού; \|  \|  \|  \| \| 43 \| Πως ονομάζουμε το αντικείμενο με το οποίο κάνουμε μυτερό ένα μολύβι; \|  \|  \|  \| \| 44 \| Πως λέγεται το λευκό αντικείμενο με το οποίο ο δάσκαλος γράφει στον πίνακα; \|  \|  \|  \| \| 45 \| Πως λέμε την τεχνητή οδοντοστοιχία που φορά κάποιος που δεν έχει δόντια: \|  \|  \|  \| \| 46 \| Πώς ονομάζεται η άνοδος της θερμοκρασίας του σώματος, όταν κάποιος είναι ασθενής; \|  \|  \|  \| \| 47 \| Πως λέγεται ο αγωγός στη στέγη ενός σπιτιού από τον οποίο βγαίνει ο καπνός από το τζάκι; \|  \|  \|  \| \| 48 \| Πώς λέγονται τα μικρά νεογέννητα σκυλιά; \|  \|  \|  \| \|  \| **Eρωτήσεις** \| **Σωστό** \| **Λάθος** \| **Καμιά Απάντηση** \| \| 49 \| Πως λέγεται το μαγειρικό σκεύος στο οποίο ετοιμάζουμε με λάδι ή βούτυρο αυγά μάτια; \|  \|  \|  \| \| 50 \| Πώς λέμε το πρώτο γεύμα της ημέρας; \|  \|  \|  \|   **Λύσεις**   1. Γενέθλια 2. Ζέβρα 3. Διαιτητής 4. Καγκουρό 5. Κουκούλι 6. Στάχτη 7. Σύριγγα 8. Αράχνη 9. Απολυτήριο 10. Πλυντήριο 11. Μελάνι 12. Σφυρί 13. Κόλλα 14. Τρύπα 15. Δεκέμβρης 16. Καταιγίδα 17. Πορσελάνη 18. Χορτοφάγος 19. Μαέστρος 20. Κάκτος 21. Κήρυγμα 22. Λάβα 23. Απολίθωμα 24. Γάτα 25. Άμβωνας 26. Ισημερινός 27. Κλωστή 28. Κεφαλαίο 29. Φόροι 30. Ενέχυρο 31. Νυστέρι 32. Τραγιάσκα 33. Καρφίτσα 34. Δικαστής 35. Κληρονομιά 36. Συγγραφέας 37. Ηθοποιός 38. Τσιγκουνιά 39. Μάρτυρας 40. Γλάστρα 41. Άμπωτη 42. Χαίτη 43. Ξύστρα 44. Κιμωλία 45. Μασέλα 46. Πυρετός 47. Kαπνοδόχος 48. Κουτάβι 49. Τηγάνι 50. Πρωϊνό |
| --- | --- | --- | --- | --- | --- | --- | --- | --- | --- | --- | --- | --- | --- | --- | --- | --- | --- | --- | --- | --- | --- | --- | --- | --- | --- | --- | --- | --- | --- | --- | --- | --- | --- | --- | --- | --- | --- | --- | --- | --- | --- | --- | --- | --- | --- | --- | --- | --- | --- | --- | --- | --- | --- | --- | --- | --- | --- | --- | --- | --- | --- | --- | --- | --- | --- | --- | --- | --- | --- | --- | --- | --- | --- | --- | --- | --- | --- | --- | --- | --- | --- | --- | --- | --- | --- | --- | --- | --- | --- | --- | --- | --- | --- | --- | --- | --- | --- | --- | --- | --- | --- | --- | --- | --- | --- | --- | --- | --- | --- | --- | --- | --- | --- | --- | --- | --- | --- | --- | --- | --- | --- | --- | --- | --- | --- | --- | --- | --- | --- | --- | --- | --- | --- | --- | --- | --- | --- | --- | --- | --- | --- | --- | --- | --- | --- | --- | --- | --- | --- | --- | --- | --- | --- | --- | --- | --- | --- | --- | --- | --- | --- | --- | --- | --- | --- | --- | --- | --- | --- | --- | --- | --- | --- | --- | --- | --- | --- | --- | --- | --- | --- | --- | --- | --- | --- | --- | --- | --- | --- | --- | --- | --- | --- | --- | --- | --- | --- | --- | --- | --- | --- | --- | --- | --- | --- | --- | --- | --- | --- | --- | --- | --- | --- | --- | --- | --- | --- | --- | --- | --- | --- | --- | --- | --- | --- | --- | --- | --- | --- | --- | --- | --- | --- | --- | --- | --- | --- | --- | --- | --- | --- | --- | --- | --- | --- | --- | --- | --- | --- | --- | --- | --- | --- | --- | --- | --- | --- | --- | --- | --- | --- | --- | --- | --- | --- | --- | --- | --- | --- | --- |

**
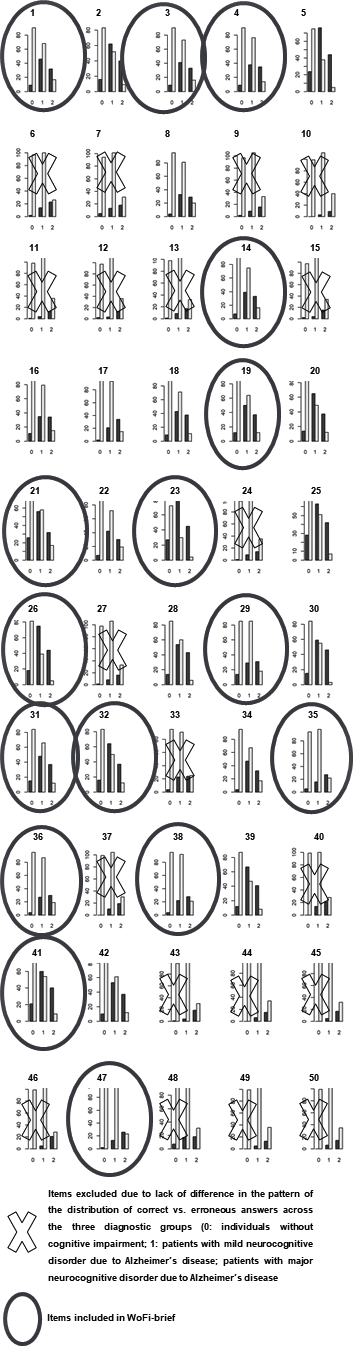
Figure 1S**

Distribution of correct vs. erroneous answers to each item of the Greek version of the Test for Finding Word retrieval deficits (WoFi) across the three diagnostic groups, i.e. individuals without cognitive impairment (0), patients with mild neurocognitive disorder due to Alzheimer’s disease (1) and patients with major neurocognitive disorder due to Alzheimer’s disease (2). Encircled are the items that are included in the brief version of WoFi (WoFi-brief)

**False answers**

0

**Correct answers**

**Table 2S** Τhe brief version of the Test for Finding Word retrieval deficits (WoFi-brief)

| **Σύντομη Δοκιμασία Εύρεσης Λέξεων**  Όνομα: Ηλικία:  Φύλο:  Έτη εκπαίδευσης: Μητρική γλώσσα:  Επάγγελμα: Hμερομηνία:   \|  \| **Ερώτηση** \| **Σωστό** \| **Λάθος** \| **Καμιά Απάντηση** \| \| --- \| --- \| --- \| --- \| --- \| \| 1 \| Πως λέμε την ημέρα που γινόμαστε κατά ένα χρόνο μεγαλύτεροι; \|  \|  \|  \| \| 2 \| Πως λέγεται στα ελληνικά ο άνθρωπος που επιβλέπει και ρυθμίζει τη διεξαγωγή ενός αγώνα σύμφωνα με ορισμένους κανονισμούς; \|  \|  \|  \| \| 3 \| Πως λέγεται το μαρσιποφόρο ζώο που ζει στην Αυστραλία; \|  \|  \|  \| \| 4 \| Πως λέγεται η άκρη της βελόνας μέσα από την οποία περνάμε την κλωστή; \|  \|  \|  \| \| 5 \| Πως ονομάζουμε το άτομο που διευθύνει μια ορχήστρα; \|  \|  \|  \| \| 6 \| Πως λέμε τον λόγο που βγάζει ένας παπάς στην εκκλησία; \|  \|  \|  \| \| 7 \| Πως ονομάζουμε το υπόλειμμα του σώματος ενός οργανισμού που διατηρήθηκε ανάμεσα σε πετρώματα παλαιότερων γεωλογικών περιόδων; \|  \|  \|  \| \| 8 \| Πως λέμε την νοητή γραμμή που χωρίζει τη γη στο βόρειο και στο νότιο ημισφαίριο; \|  \|  \|  \| \| 9 \| Πως λέγονται τα χρήματα που καταβάλουμε υποχρεωτικά στο κράτος για την κάλυψη δημοσίων δαπανών ή άλλων αναγκών; \|  \|  \|  \| \| 10 \| Πως ονομάζουμε το κοφτερό μαχαίρι που χρησιμοποιούν οι χειρουργοι; \|  \|  \|  \| \| 11 \| Πως λέγεται το καπέλο/ κασκέτο που φορούν σήμερα συνήθως ηλικιωμένοι άνδρες και παλαιότερα οι εργάτες; \|  \|  \|  \| \|  \| **Ερώτηση** \| **Σωστό** \| **Λάθος** \| **Καμιά Απάντηση** \| \| 12 \| Πως λέγεται η περιουσία που λαμβάνει κάποιος μετά τον θάνατο των γονιών του; \|  \|  \|  \| \| 13 \| Πως λέγεται αυτός που γράφει ένα βιβλίο ή ένα μυθιστόρημα; \|  \|  \|  \| \| 14 \| Πως ονομάζουμε την υπερβολική οικονομία χρημάτων; \|  \|  \|  \| \| 15 \| Πως λέγεται το αντίθετο της παλίρροιας; \|  \|  \|  \| \| 16 \| Πως λέγεται ο αγωγός στη στέγη ενός σπιτιού από τον οποίο βγαίνει ο καπνός από το τζάκι; \|  \|  \|  \| |
| --- | --- | --- | --- | --- | --- | --- | --- | --- | --- | --- | --- | --- | --- | --- | --- | --- | --- | --- | --- | --- | --- | --- | --- | --- | --- | --- | --- | --- | --- | --- | --- | --- | --- | --- | --- | --- | --- | --- | --- | --- | --- | --- | --- | --- | --- | --- | --- | --- | --- | --- | --- | --- | --- | --- | --- | --- | --- | --- | --- | --- | --- | --- | --- | --- | --- | --- | --- | --- | --- | --- | --- | --- | --- | --- | --- | --- | --- | --- | --- | --- | --- | --- | --- | --- | --- | --- | --- | --- | --- | --- |
